# ^89^Zr-PET imaging to predict tumor uptake of ^177^Lu-NNV003 anti-CD37 radioimmunotherapy in mouse models of B cell lymphoma

**DOI:** 10.1038/s41598-022-10139-6

**Published:** 2022-04-15

**Authors:** Danique Giesen, Marjolijn N. Lub-de Hooge, Marcel Nijland, Helen Heyerdahl, Jostein Dahle, Elisabeth G. E. de Vries, Martin Pool

**Affiliations:** 1 Department of Medical Oncology, University Medical Center Groningen, P.O. Box 30.001, 9700 RB Groningen, The Netherlands; 2 Department of Clinical Pharmacy and Pharmacology, University Medical Center Groningen, Groningen, The Netherlands; 3 Department of Nuclear Medicine and Molecular Imaging, University Medical Center Groningen, Groningen, The Netherlands; 4 Department of Hematology, University Medical Center Groningen, Groningen, The Netherlands; 5 Nordic Nanovector ASA, Oslo, Norway; 6 Department of Clinical Pharmacy and Toxicology, Leiden University Medical Center, Leiden, The Netherlands

**Keywords:** Pharmacokinetics, Positron-emission tomography, Cancer imaging, Cancer therapy, Haematological cancer

## Abstract

[^177^Lu]Lu-DOTA-NNV003, a radioimmunoconjugate targeting CD37, is developed as novel radioimmunotherapy (RIT) treatment for patients with B cell non-Hodgkin’s lymphoma (NHL). Since patients are at risk for developing hematological toxicities due to CD37 expression on normal B cells, we aimed to develop ^89^Zr-labeled NNV003 for positron emission tomography (PET) imaging as a surrogate tool to predict [^177^Lu]Lu-DOTA-NNV003 RIT whole-body distribution and tumor uptake. NNV003 antibody was first radiolabeled with ^89^Zr. [^89^Zr]Zr-*N*-sucDf-NNV003 tumor uptake was evaluated by PET imaging of mice bearing human CD37-expressing REC1 B cell NHL or RAMOS Burkitt’s lymphoma xenograft tumors followed by ex vivo analysis. Finally, CD37-targeting of [^89^Zr]Zr-*N*-sucDf-NNV003 and [^177^Lu]Lu-DOTA-NNV003 RIT were compared. [^89^Zr]Zr-*N*-sucDf-NNV003 accumulated in REC1 tumors over time, which was not observed for non-specific, ^111^In-labeled IgG control molecule. In RAMOS tumor-bearing mice, [^89^Zr]Zr-*N*-sucDf-NNV003 tumor uptake was higher than [^111^In]In-DTPA-IgG at all tested tracer protein doses (10 µg, 25 µg and 100 µg; *P* < 0.01), further confirming [^89^Zr]Zr-*N*-sucDf-NNV003 tumor uptake is CD37-mediated. [^89^Zr]Zr-*N*-sucDf-NNV003 and [^177^Lu]Lu-DOTA-NNV003 RIT showed similar ex vivo biodistribution and tumor uptake in the RAMOS tumor model. In conclusion, [^89^Zr]Zr-*N*-sucDf-NNV003 PET imaging can serve to accurately predict CD37-targeting of [^177^Lu]Lu-DOTA-NNV003. To enable clinical implementation, we established a good manufacturing practice (GMP)-compliant production process for [^89^Zr]Zr-*N*-sucDf-NNV003.

## Introduction

Novel treatment options for patients with B cell non-Hodgkin’s lymphoma (NHL) are warranted, especially for those with poor prognosis, since large subgroups of these patients become refractory after initial CD20-based immunotherapy. Therapies directed against other targets expressed by B cells, for example leukocyte antigen CD37, may provide a therapeutic alternative. CD37-directed radioimmunotherapy (RIT) with the fully murine antibody lutetium-177 (^177^Lu; T_1/2_: 6.65 d)-lilotomab satetraxetan, was recently evaluated in a phase 1/2a study in relapsed/refractory indolent NHL patients and showed encouraging results^1–3^. Instead of a murine antibody, next-generation [^177^Lu]Lu-DOTA-NNV003 consists of a chimeric mouse-human anti-CD37 antibody (NNV003) conjugated with p-SCN-Bn-DOTA that chelates the β^–^ emitting radionuclide ^177^Lu^4^.

CD37 is a highly glycosylated transmembrane protein selectively expressed by normal B cells and the majority of B cell lymphomas and is specifically of interest for RIT, since CD37 receptor-antibody complexes are highly internalized in tumor cells^5,6^. In combination with ^177^Lu’s favorable physical properties for RIT, [^177^Lu]Lu-DOTA-NNV003 has a potentially improved toxicity profile. Compared to historically used radionuclides for RIT in B cell malignancies such as yttrium-90 (^90^Y; T_1/2_: 2.66 d), ^177^Lu has a relatively short β^-^‐range^7^. Also, ^177^Lu becomes intracellularly trapped in lysosomes through residualization, whereas iodine-131 (^131^I; T_1/2_: 8.02 d) diffuses passively out of cells after catabolization^8^. This may result in enhanced tumor irradiation, while sparing surrounding healthy tissues.

RIT in B cell malignancies is generally effective in only a subset of patients, while often resulting in on-target, off-tumor toxicities due to target expression on normal B cells^9^. Insight in CD37 expression may help to select patients who are more likely to respond or are at risk for developing CD37-induced hematological toxicities. Therefore, RIT could benefit from molecular imaging as a non-invasive approach to provide whole-body information on target presence and RIT distribution. Dosimetry for organs-at-risk in ^177^Lu radionuclide therapy is often calculated using single photon emission computed tomography (SPECT)/computed tomography (CT) imaging. Still, this procedure has limited sensitivity and quantitative evaluation of target-mediated uptake can be challenging^10,11^.

**Figure 1:**
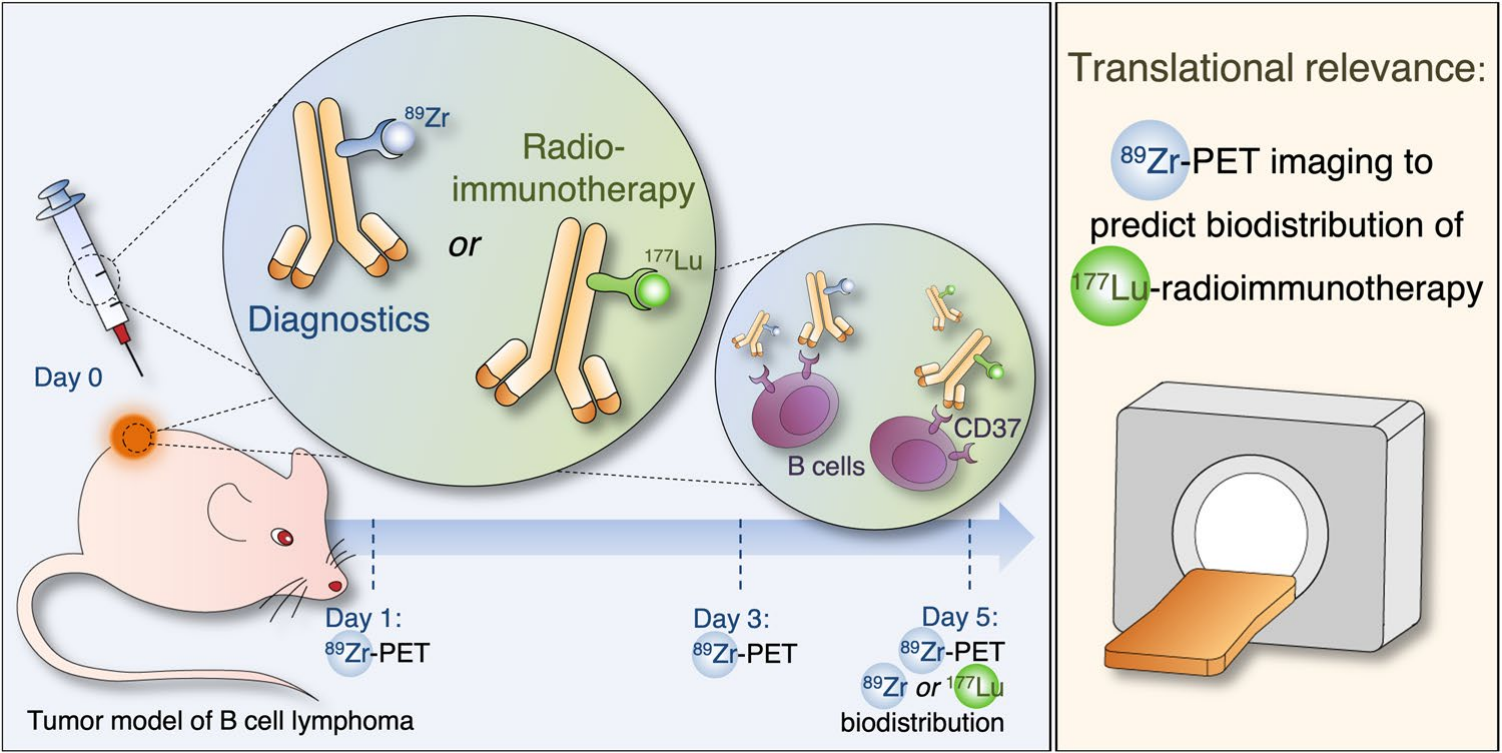
Schematic overview of [^89^Zr]Zr-*N*-sucDf-NNV003 anti-CD37 PET imaging in mouse models of B cell lymphoma and its potential implementations. We aimed to develop [^89^Zr]Zr-*N*-sucDf-NNV003 as a surrogate image tool for the assessment of [^177^Lu]Lu-DOTA-NNV003 RIT whole-body distribution and tumor uptake. [^89^Zr]Zr-*N*-sucDf-NNV003 pre-therapy PET imaging in patients with B cell NHL may help to identify those more likely to respond. Furthermore, as a surrogate imaging tool, [^89^Zr]Zr-*N*-sucDf-NNV003 may aid in optimizing [^177^Lu]Lu-DOTA-NNV003 RIT dose-regimens and post-therapy response evaluation.

We aimed to develop a surrogate image tool for the assessment of [^177^Lu]Lu-DOTA-NNV003 RIT whole-body distribution and tumor uptake, which could aid its clinical development and use (Fig. 1). The positron emission tomography (PET) radioisotope zirconium-89 (^89^Zr) is particularly suited for antibody imaging, as it yields high sensitivity and accurate quantification, while its physical half-life of 3.27 d matches the time that antibodies need for tumor accumulation. Studies in patients and mice showed the utility of ^89^Zr-PET imaging to predict the distribution of therapeutic radionuclides such as ^177^Lu and ^90^Y coupled to antibodies^12–14^.

To assess whether [^89^Zr]Zr-*N*-sucDf-NNV003 PET imaging can serve to predict [^177^Lu]Lu-DOTA-NNV003 RIT biodistribution, we evaluated their whole-body distribution and tumor-targeting properties in mice bearing human B cell lymphomas. Furthermore, a good manufacturing practice (GMP)-compliant production process was established for [^89^Zr]Zr-*N*-sucDf-NNV003 to enable administration to patients.

## Results

### Development and quality control of ^89^Zr-labeled NNV003

For in vivo studies, NNV003 was conjugated to tetrafluorphenol-*N*-succinyldesferal (TFP-N-sucDf) followed by radiolabeling with ^89^Zr. NNV003 was incubated with increasing molar ratios of TFP-*N*-sucDf, which resulted in approximately 60% conjugation efficiency (Supplementary Fig. S1A). NNV003-*N*-sucDf intermediate product was obtained with a mean yield of 54% (Supplementary Fig. S1B). To enable ^89^Zr-chelation by TFP-*N*-sucDf, protective Fe(III) must be removed from the hydroxamate groups. At least 40% Fe(III) was removed from NNV003-*N*-sucDf by incubation with EDTA (Supplementary Fig. S1C). The NNV003:TFP-*N*-sucDf ratio did not impair immunoreactivity of NNV003-*N*-sucDf to CD37-expressing RAMOS cells, as the mean immunoreactive fractions (IRF) were similar for all tested ratios and all higher than 0.8 (Supplementary Fig. S1D). NNV003-*N*-sucDf intermediate product was obtained with ≥ 95% purity and no aggregation or fragmentation was observed (Supplementary Fig. S1E). NNV003 conjugated to ~ 1.2 TFP-*N*-sucDf chelators per antibody consistently bound 500 MBq ^89^Zr per mg (radiochemical purity; RCP ≥ 95%) with preserved immunoreactivity and is therefore considered most optimal for in vivo studies (Supplementary Fig. S1F).

### [^89^Zr]Zr-*N*-sucDf-NNV003 PET imaging and biodistribution in REC1 tumor-bearing mice

**Figure 2:**
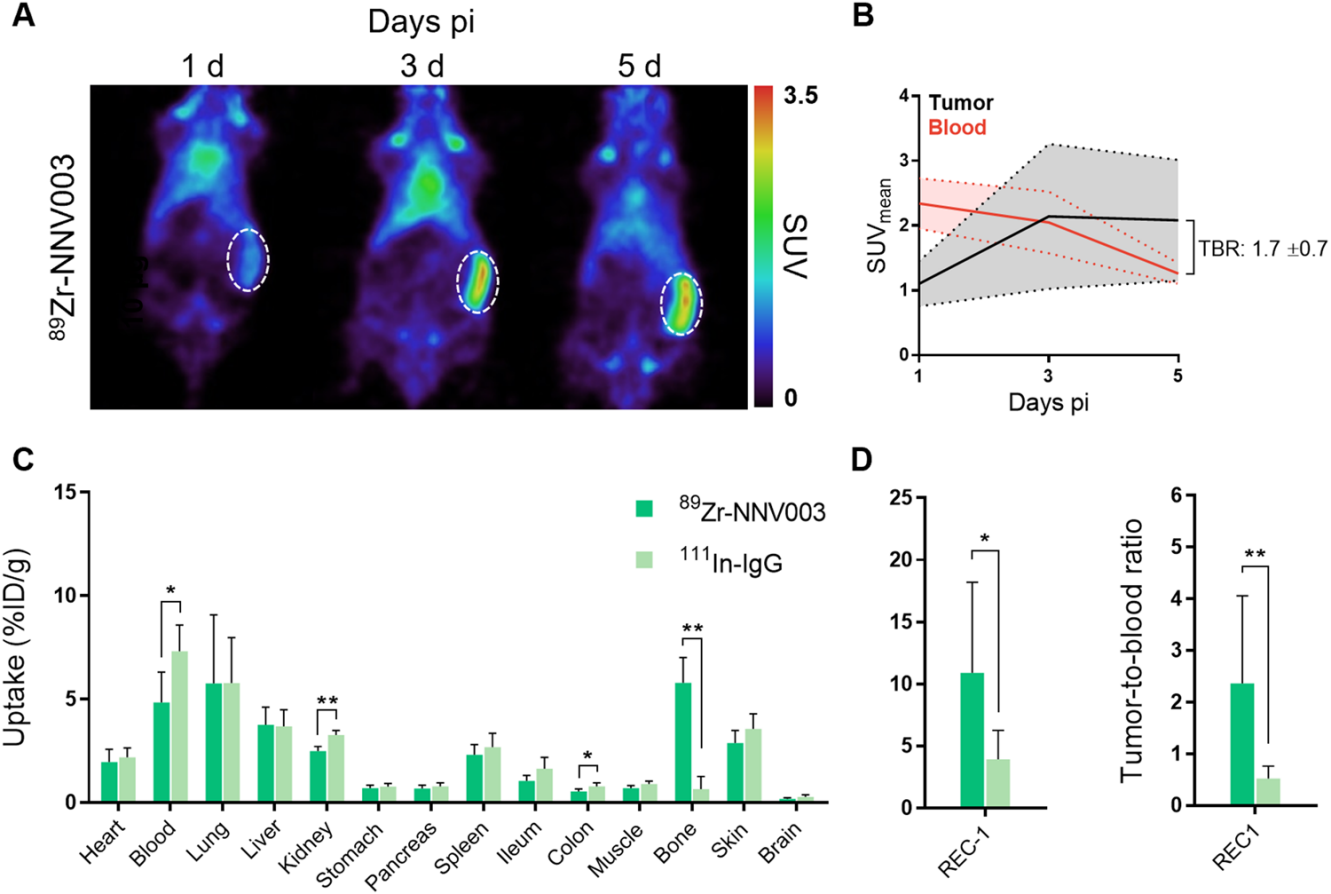
[^89^Zr]Zr-*N*-sucDf-NNV003 PET imaging and ex vivo biodistribution in REC1 xenografted mice. (**A**) Representative coronal PET images of 10 µg [^89^Zr]Zr-*N*-sucDf-NNV003 biodistribution in REC1 tumor-bearing mice at 1, 3 and 5 days (d) pi. [^89^Zr]Zr-*N*-sucDf-NNV003 uptake is presented as standardized uptake value (SUV). The dashed circle indicates REC1 tumor. (**B**) Quantification of [^89^Zr]Zr-*N*-sucDf-NNV003 uptake in REC1 tumor and blood pool activity at 1, 3 and 5 days pi. [^89^Zr]Zr-*N*-sucDf-NNV003 uptake is presented as mean standardized uptake value (SUV_mean_). TBR indicates tumor-to-blood ratio at day 5 pi. (**C**) Ex vivo biodistribution results of 10 µg [^89^Zr]Zr-*N*-sucDf-NNV003 versus [^111^In]In-DTPA-IgG control in REC1 tumor-bearing mice at 5 days pi. Tracer uptake per organ is presented as percentage of injected radioactivity dose per gram tissue (%ID/g). (**D**) Left: Ex vivo REC1 tumor uptake of 10 µg [^89^Zr]Zr-*N*-sucDf-NNV003 versus [^111^In]In-DTPA-IgG control at day 5 pi. Tumor uptake of tracer is presented as %ID/g. Right: Ex vivo tumor-to-blood ratio of 10 µg [^89^Zr]Zr-*N*-sucDf-NNV003 versus [^111^In]In-DTPA-IgG control in REC1 tumor-bearing mice at day 5 pi. Data in (**B**–**D**) is shown as mean ± standard deviation (SD). **P < 0.01, *P < 0.05, *ns* not significant.

[^89^Zr]Zr-*N*-sucDf-NNV003 whole-body distribution and tumor-targeting were evaluated in BALB/c nude mice bearing CD37-expressing human REC1 B cell NHL xenograft tumors. PET imaging revealed [^89^Zr]Zr-*N*-sucDf-NNV003 tumor uptake increased between day 1 and day 5 pi, whereas blood pool activity decreased (Fig. 2A). PET quantification showed the highest tumor uptake with mean standardized uptake value (SUV_mean_) of 2.1 (± 0.9) at both 3 and 5 days pi and highest tumor-to-blood ratio of 1.7 (± 0.7) at day 5 pi (Fig. 2B).

Ex vivo biodistribution in healthy tissues was similar for [^89^Zr]Zr-*N*-sucDf-NNV003 and non-specific, indium-111 (^111^In; T_1/2_: 2.80 d)-labeled IgG control molecule, however [^89^Zr]Zr-*N*-sucDf-NNV003 demonstrated lower activity in the blood pool, kidneys and colon, whereas uptake in bone was higher compared to [^111^In]In-DTPA-IgG at day 5 pi (Fig. 2C). Bone uptake is common for ^89^Zr-labeled molecules, as they are metabolized in vivo and unbound ^89^Zr has a high affinity for hydroxyapatite^15^. Levels similar to the [^89^Zr]Zr-*N*-sucDf-NNV003 uptake in bone were found in mouse models for other ^89^Zr-labeled antibodies^16,17^. For this reason, potential instability of [^89^Zr]Zr-*N*-sucDf-NNV003 linker chelation is considered to be limited. Ex vivo tumor uptake and tumor-to-blood ratio were higher for [^89^Zr]Zr-*N*-sucDf-NNV003 compared to [^111^In]In-DTPA-IgG (10.9 vs. 3.9%ID/g; *P* < 0.05 and 2.4 vs. 0.5; *P* < 0.01) (Fig. 2D), indicating [^89^Zr]Zr-*N*-sucDf-NNV003 tumor uptake is CD37-mediated. Furthermore, [^111^In]In-DTPA-IgG tumor-to-blood ratio was lower than 1, meaning tumor uptake is not target-mediated. [^111^In]In-DTPA-IgG can therefore be considered a suitable control molecule.

### Dose- and CD37-dependent uptake in RAMOS tumor-bearing mice

**Figure 3:**
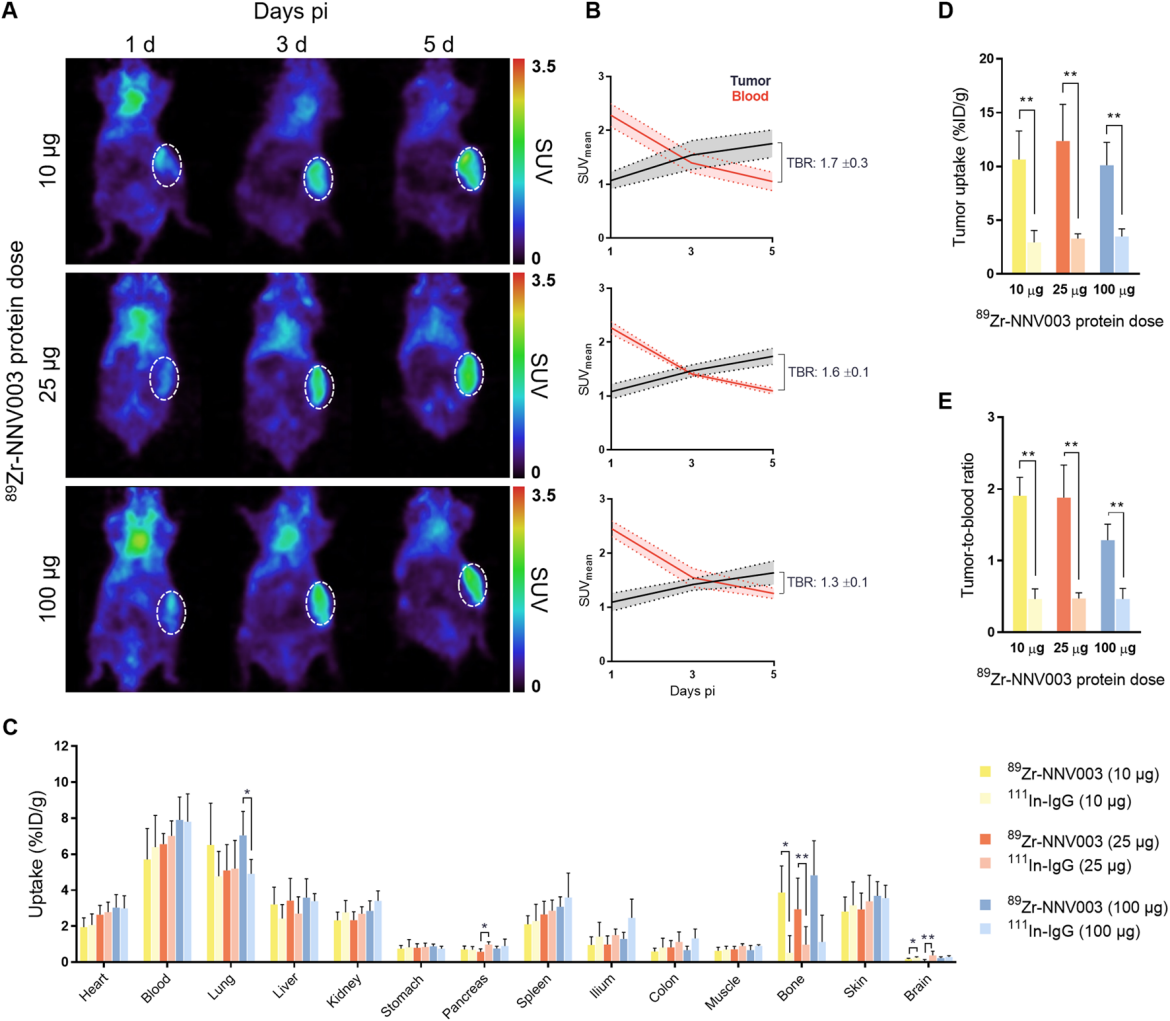
[^89^Zr]Zr-*N*-sucDf-NNV003 PET imaging and ex vivo biodistribution at increasing protein dose in RAMOS xenografted mice. (**A**) Representative coronal PET images of 10, 25 and 100 µg [^89^Zr]Zr-*N*-sucDf-NNV003 biodistribution in RAMOS tumor-bearing mice at 1, 3 and 5 days (d) pi. [^89^Zr]Zr-*N*-sucDf-NNV003 uptake is presented as SUV. RAMOS tumor is indicated by a white, dashed circle. (**B**) Quantification of [^89^Zr]Zr-*N*-sucDf-NNV003 uptake in RAMOS tumor and blood pool activity for 10, 25 and 100 µg protein doses at 1, 3 and 5 days pi. [^89^Zr]Zr-*N*-sucDf-NNV003 uptake is presented as SUV_mean_. TBR indicates tumor-to-blood ratio at day 5 pi. (**C**) Ex vivo biodistribution results of [^89^Zr]Zr-*N*-sucDf-NNV003 versus [^111^In]In-DTPA-IgG control dose-escalation in RAMOS tumor-bearing mice at 5 days pi. Tracer uptake per organ is presented as %ID/g. (**D**) Ex vivo RAMOS uptake of [^89^Zr]Zr-*N*-sucDf-NNV003 versus [^111^In]In-DTPA-IgG control for 10 µg, 25 µg and 100 µg protein doses at 5 days pi. Tumor uptake is presented as %ID/g. (**E**) Ex vivo tumor-to-blood ratios of [^89^Zr]Zr-*N*-sucDf-NNV003 versus [^111^In]In-DTPA-IgG control in RAMOS tumor bearing mice for 10 µg, 25 µg and 100 µg protein doses at 5 days pi. Data in (**B**–**E**) is shown as mean ± SD. **P < 0.01, *P < 0.05, *ns* not significant.

To study CD37-mediated tumor uptake, we evaluated [^89^Zr]Zr-*N*-sucDf-NNV003 biodistribution at three total protein dose levels in human RAMOS Burkitt’s lymphoma tumor-bearing mice. A radiolabeled antibody dose of 10 μg [^89^Zr]Zr-*N*-sucDf-NNV003 or [^111^In]In-DTPA-IgG was supplemented with 0, 15 or 90 µg of unlabeled NNV003 or IgG to obtain total protein doses of 10 µg, 25 µg and 100 µg for each tracer. PET quantification showed [^89^Zr]Zr-*N*-sucDf-NNV003 accumulation in CD37-expressing RAMOS tumors for all tested protein doses, with the highest tumor-to-blood ratios found for day 5 pi (Fig. 3A). Comparable tumor uptake was observed for 10, 25 and 100 µg [^89^Zr]Zr-*N*-sucDf-NNV003, with SUV_mean_ of 1.8 (± 0.3), 1.7 (± 0.1) and 1.6 (± 0.2) respectively at day 5 pi (Fig. 3B). The highest tumor-to-blood ratio (1.7 ± 0.3) was observed for 10 µg [^89^Zr]Zr-*N*-sucDf-NNV003 at day 5 pi.

No relevant differences in ex vivo biodistribution of [^89^Zr]Zr-*N*-sucDf-NNV003 and [^111^In]In-DTPA-IgG were observed, except for uptake in bone and tumor (Fig. 3C). [^89^Zr]Zr-*N*-sucDf-NNV003 bone uptake demonstrated some variation between 10 µg, 25 µg and 100 µg dose groups, potentially due to slight differences in radiochemical purity, which varied from 95 to 98%. Also, [^89^Zr]Zr-*N*-sucDf-NNV003 distribution to healthy tissues was not affected by the addition of unlabeled antibody dose. Ex vivo [^89^Zr]Zr-*N*-sucDf-NNV003 tumor uptake was higher compared to [^111^In]In-DTPA-IgG for all protein dose groups, confirming that [^89^Zr]Zr-*N*-sucDf-NNV003 tumor uptake is CD37-mediated (Fig. 3D). Furthermore, tumor uptake was comparable for 10, 25 and 100 µg [^89^Zr]Zr-*N*-sucDf-NNV003, with 10.6 (± 2.7), 12.4 (± 3.4) and 10.1 (± 2.1) %ID/g respectively, indicating CD37 is not saturated in tumor cells by addition of unlabeled antibody at these protein doses. The highest [^89^Zr]Zr-*N*-sucDf-NNV003 tumor-to-blood ratio of 1.9 (± 0.3) and 1.9 (± 0.5) was observed for the 10 and 25 µg total protein doses, compared to 1.3 (± 0.2) found for 100 µg [^89^Zr]Zr-*N*-sucDf-NNV003, suggesting CD37 in RAMOS tumors may be saturated by [^89^Zr]Zr-*N*-sucDf-NNV003 at higher protein doses (Fig. 3E). [^89^Zr]Zr-*N*-sucDf-NNV003 tumor uptake was the highest in the 25 µg dose group compared to [^111^In]In-DTPA-IgG (12.4 vs. 3.3%ID/g; *P* < 0.01).

**Figure 4:**
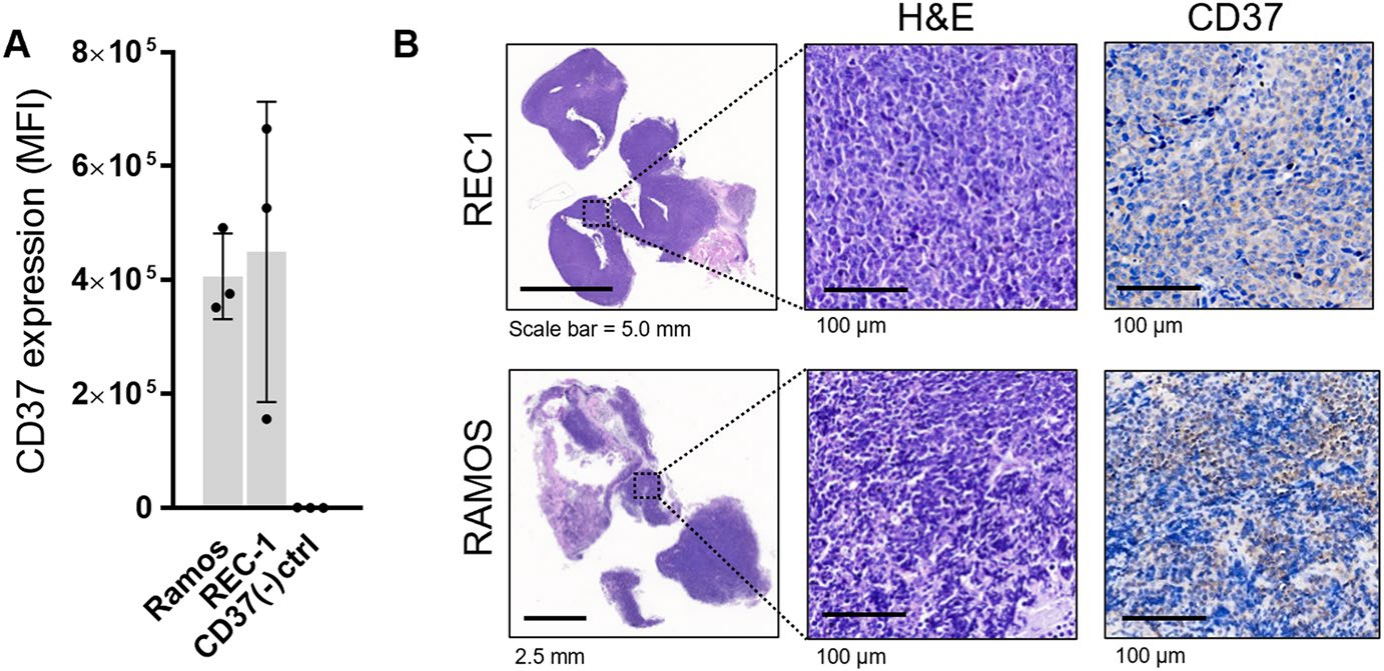
CD37 expression in RAMOS and REC-1 tumors. (**A**) In vitro CD37 expression in REC1 and RAMOS tumors determined by flow cytometry. CD37 expression is presented as mean fluorescent intensity (MFI). HCC827 lung adenocarcinoma cells were used as negative control. Data is shown as mean ± SD. (**B**) Hematoxylin and eosin staining and CD37 immunohistochemistry on formalin-fixed, paraffin-embedded REC1 and RAMOS tumor tissue sections. Representative tumors are shown.

[^89^Zr]Zr-*N*-sucDf-NNV003 tumor uptake was comparable for REC1 and RAMOS tumors, with both tumor types expressing similar levels of CD37 as measured by flow cytometry and immunohistochemistry (Fig. 4).

**Figure 5:**
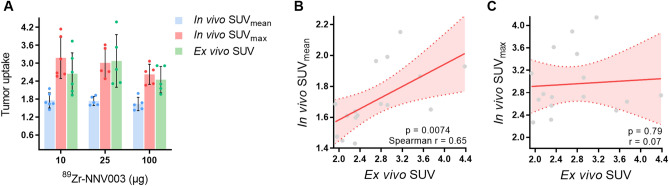
Correlation between in vivo (PET) and ex vivo (gammacounter) [^89^Zr]Zr-*N*-sucDf-NNV003 tumor uptake. (**A**) In vivo [^89^Zr]Zr-*N*-sucDf-NNV003 tumor uptake expressed as SUV_mean_ and SUV_max_ versus ex vivo tumor uptake. Ex vivo SUV was calculated by correcting %ID/g for injected dose and body weight. Data is shown as mean ± SD. (**B**) Correlation between in vivo SUV_mean_ and (**C**) SUV_max_ versus ex vivo tumor uptake. Data is shown as mean with fitted regression curve ± 95% confidence interval (CI).

Lastly, we compared tumor uptake determined by PET quantification with ex vivo tumor uptake to confirm if [^89^Zr]Zr-*N*-sucDf-NNV003 PET imaging accurately visualizes tumor uptake. Ex vivo [^89^Zr]Zr-*N*-sucDf-NNV003 tumor uptake expressed as SUV_mean_ correlated with PET-derived tumor SUV_mean_, but not SUV_max_, which can be explained by the fact that SUV_max_ represents the highest voxel and may not reflect whole-tumor uptake (Fig. 5).

### Comparison of [^89^Zr]Zr-***N***-sucDf-NNV003 and [^177^Lu]Lu-DOTA-NNV003 RIT biodistribution in the RAMOS tumor model

**Figure 6:**
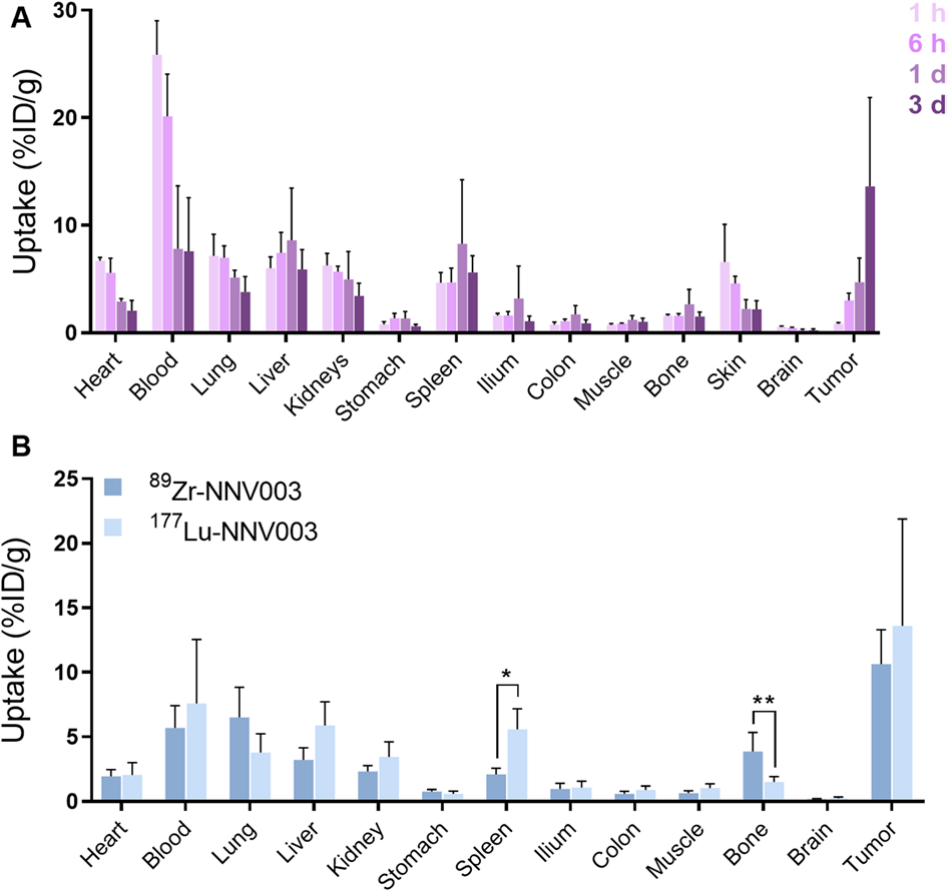
[^177^Lu]Lu-DOTA-NNV003 biodistribution in RAMOS xenografted mice and comparison with [^89^Zr]Zr-*N*-sucDf-NNV003. (**A**) Ex vivo biodistribution results of [^177^Lu]Lu-DOTA-NNV003 in RAMOS-tumor bearing mice at 1 h (h), 6 h, 1 day (d) and 3 days pi. Tracer uptake per organ is presented as %ID/g. Left: [^177^Lu]Lu-DOTA-NNV003 uptake in healthy tissues. Right: [^177^Lu]Lu-DOTA-NNV003 uptake in tumor. (**B**) [^89^Zr]Zr-*N*-sucDf-NNV003 (500 MBq/mg) and [^177^Lu]Lu-DOTA-NNV003 (50–90 MBq/mg) ex vivo biodistribution results at 5 days pi and 3 days pi respectively. Uptake is presented as %ID/g. Data is shown as mean ± SD. **P < 0.01, *P < 0.05, *ns* not significant.

Next, we evaluated [^89^Zr]Zr-*N*-sucDf-NNV003 as a surrogate for [^177^Lu]Lu-DOTA-NNV003 RIT whole-body distribution in RAMOS tumor-bearing mice. Ex vivo tissue analysis showed decreasing [^177^Lu]Lu-DOTA-NNV003 activity in the blood pool between 1 h to 3 days pi, which coincided with decreasing uptake in well-perfused organs such as heart, lung and kidney (Fig. 6A). [^177^Lu]Lu-DOTA-NNV003 uptake in RAMOS tumors increased over time with the highest uptake of 13.6 ± 8.3%ID/g found at 3 days pi. Blood pool activity of [^89^Zr]Zr-*N*-sucDf-NNV003 at 5 days pi was comparable to [^177^Lu]Lu-DOTA-NNV003 at 3 days pi (5.7 ± 1.7%ID/g versus 7.6 ± 5.0%ID/g), validating a relevant comparison of both tracers’ ex vivo biodistribution results.

Comparison of ex vivo biodistribution results revealed highest uptake of [^89^Zr]Zr-*N*-sucDf-NNV003 and [^177^Lu]Lu-DOTA-NNV003 in tumor compared to normal tissues (Fig. 6B). Also, similar uptake was found for [^89^Zr]Zr-*N*-sucDf-NNV003 and [^177^Lu]Lu-DOTA-NNV003 in RAMOS tumors (10.6 vs. 13.6%ID/g; *P* = 0.48), indicating tumor-targeting is not affected by chelator or radioisotope (TFP-N-sucDf for ^89^Zr versus p-SCN-Bn-DOTA for ^177^Lu). Furthermore, [^177^Lu]Lu-DOTA-NNV003 and [^89^Zr]Zr-*N*-sucDf-NNV003 showed similar biodistribution in healthy tissues, except for bone and spleen. Bone uptake was higher for [^89^Zr]Zr-*N*-sucDf-NNV003, which is common for ^89^Zr-labeled antibodies, as discussed previously^15^. In patients, tracer uptake in red bone marrow can be clearly separated from bone uptake on PET. Therefore, toxicity from myelosuppression can be potentially recognized by high [^89^Zr]Zr-*N*-sucDf-NNV003 uptake in red bone marrow.

Higher spleen uptake was observed for [^177^Lu]Lu-DOTA-NNV003 compared to [^89^Zr]Zr-*N*-sucDf-NNV003 (5.6 vs. 2.1%ID/g; *P* < 0.05). Murine CD37 is expected to be expressed on B cells present in the spleen, however NNV003 is not cross-reactive with murine CD37. Furthermore, [^89^Zr]Zr-*N*-sucDf-NNV003 spleen uptake was similar to [^111^In]In-DTPA-IgG, indicating this uptake is not CD37-mediated. In mice, the spleen plays an important role in antibody pharmacokinetics due to its high blood flow and loose capillaries, but also due to the expression of Fcγ receptors. Therefore, differences in tracer spleen uptake are easily induced by slight differences in the immune status of mice^18^.

### Clinical-grade [^89^Zr]Zr-***N***-sucDf-NNV003 for patient studies

To enable PET imaging in patients, we developed and characterized the production of clinical-grade [^89^Zr]Zr-*N*-sucDf-NNV003 (Supplementary Fig. S2). Quality control for three individual batches of NNV003-*N*-sucDf intermediate product and [^89^Zr]Zr-*N*-sucDf-NNV003 final product was according to specifications, indicating a robust manufacturing process (Supplementary Table S1). NNV003-*N*-sucDf intermediate product demonstrated stability up to 6 months (Supplementary Table S2). Therefore, NNV003-*N*-sucDf shelf-life is currently set at 6 months, and may be extended if future stability time points remain within specifications. Furthermore, [^89^Zr]Zr-*N*-sucDf-NNV003 final product was stable up to 96 h when stored at 2–8 °C and up to 4 h when prepared in the syringe (Supplementary Table S3).

## Discussion

Our study demonstrates the potential of using [^89^Zr]Zr-*N*-sucDf-NNV003 PET imaging to predict whole-body distribution and tumor uptake of [^177^Lu]Lu-DOTA-NNV003 RIT. We showed that [^89^Zr]Zr-*N*-sucDf-NNV003 accumulated in REC1 and RAMOS tumor tissues in a CD37-dependent manner, resulting in high tumor-to-blood ratios. Furthermore, [^89^Zr]Zr-*N*-sucDf-NNV003 biodistribution and tumor-targeting were similar to [^177^Lu]Lu-DOTA-NNV003 RIT.

^177^Lu-lilotomab satetraxetan, the murine version of [^177^Lu]Lu-DOTA-NNV003, is currently in phase II for patients with anti-CD20 refractory follicular lymphoma and has previously been studied in phase I/IIa clinical trials in patients with relapsed, CD37-positive, indolent and aggressive NHL (ClinicalTrials.gov Identifier: NCT01796171, NCT02658968)^3^. These studies showed high RIT uptake in tumors, but also in red bone marrow, liver, spleen, and kidneys^9,10^. This may be explained by CD37 expression on mature, normal B cells in these tissues. Predosing with unlabeled lilotomab significantly reduced the absorbed radiation dose in healthy CD37-expressing tissues due to ^177^Lu-lilotomab satetraxetan^9^. Also, ^177^Lu-lilotomab satetraxetan dosimetry, biodistribution, and tumor targeting were improved by lilotomab predosing compared with rituximab predosing or no predosing^3^. Informing clinicians on whether tumors are effectively targeted by [^177^Lu]Lu-DOTA-NNV003 and gaining insight into the amount of cold antibody dose required to saturate CD37 expression in healthy tissues are essential to optimize future [^177^Lu]Lu-DOTA-NNV003 RIT dose-regimens. We showed that [^89^Zr]Zr-*N*-sucDf-NNV003 PET imaging can serve as a surrogate for [^177^Lu]Lu-DOTA-NNV003 RIT whole-body distribution and represents a potentially attractive tool to assess distribution to tumors and healthy tissues.

The combined approach of RIT and diagnostics such as molecular imaging may support precise cancer therapy in both palliative and curative settings^19^. SPECT/CT imaging was routinely used for assessing biodistribution and dosimetry of ^90^Y- and ^131^I-based RIT antibodies in the early 2000s^20,21^, but low quantities of γ-photons emitted by ^177^Lu complicate quantification. As a therapeutic agent, even low dose pre-treatment imaging of ^177^Lu may result in local toxicity, while this risk is limited for the low energy β^+^-rays of PET radioisotopes. In this respect, gallium-68 (^68^Ga)/^177^Lu is a commonly used theranostic pair for studying receptor expression or drug distribution. However, ^68^Ga, given its relatively short physical half-life of 67.6 min, provides no insight in internalizing properties of an antibody. As internalization is an essential factor for both efficacy and toxicity of ^177^Lu-based RIT agents, longer-lived PET radioisotopes such as ^89^Zr may better reflect ^177^Lu RIT in vivo behavior.

In a recent study, response at the tumor lesion level after treatment with ^177^Lu-lilotomab satetraxetan was evaluated by FDG PET/CT and did not correlate with tumor-absorbed dose^11^. They hypothesized that the combination regimen of radiolabeled and cold antibodies might preclude such a correlation. In our study, we were able to quantitatively visualize tumor uptake in the presence of unlabeled antibody using [^89^Zr]Zr-*N*-sucDf-NNV003 PET imaging. In patients with relapsed B cell NHL, a pre-therapy scan with ^89^Zr-ibritumomab tiuxetan was used to predict radiation dosimetry during ^90^Y-ibritumomab tiuxetan therapy^14^. Importantly, ^89^Zr-ibritumomab tiuxetan whole-body distribution was not affected by simultaneous ^90^Y-ibritumomab tiuxetan therapy. These findings emphasize the potential of an [^89^Zr]Zr-*N*-sucDf-NNV003 pre-therapy scan to predict CD37-targeting by [^177^Lu]Lu-DOTA-NNV003 RIT, thereby allowing for patient stratification. Furthermore, a post-therapy [^89^Zr]Zr-*N*-sucDf-NNV003 scan may inform on therapy response by evaluating CD37-mediated tumor uptake after [^177^Lu]Lu-DOTA-NNV003 RIT.

Next-generation anti-CD37 RIT antibody [^177^Lu]Lu-DOTA-NNV003, based on the chimeric mouse-human antibody NNV003, was hypothesized to be less immunogenic than the fully murine ^177^Lu-lilotomab satetraxetran. After a single dose of ^177^Lu-lilotomab satetraxetan, development of human-anti-mouse-antibodies (HAMAs) was reported for seven out of 74 subjects^3^. Preclinically, [^177^Lu]Lu-DOTA-NNV003 in silico immunogenicity prediction tools revealed a lower immunogenicity potential compared to ^177^Lu-lilotumab satetraxetan^4^. These results warrant further evaluation of [^177^Lu]Lu-DOTA-NNV003 RIT in patients with CD37-expressing B cell malignancies, and [^89^Zr]Zr-*N*-sucDf-NNV003 PET imaging could assist its clinical development and use.

## Conclusion

We showed that [^89^Zr]Zr-*N*-sucDf-NNV003 PET imaging can accurately predict whole-body distribution and tumor uptake of [^177^Lu]Lu-DOTA-NNV003 therapy in B cell lymphoma xenograft models. To enable clinical implementation of this theranostic strategy, we established a GMP-compliant production process for [^89^Zr]Zr-*N*-sucDf-NNV003. [^89^Zr]Zr-*N*-sucDf-NNV003 pre-therapy PET imaging in patients with B cell NHL may help to identify those more likely to respond. Furthermore, as a surrogate imaging tool, [^89^Zr]Zr-*N*-sucDf-NNV003 may aid in optimizing [^177^Lu]Lu-DOTA-NNV003 RIT dose-regimens and post-therapy response evaluation.

## Materials and methods

### Cell lines and flow cytometry experiments

Human CD37-expressing cell lines RAMOS (Burkitt's lymphoma) and REC1 (Mantle cell lymphoma) were obtained from the American Type Culture Collection. RAMOS and REC1 cell lines were tested and authenticated in July and October 2019 respectively using short tandem repeat profiling. Cells were cultured in Roswell Park Memorial Institute (RPMI) medium, supplemented with 10% fetal calf serum (FCS) and incubated at 37 °C in a humidified atmosphere with 5% CO_2_.

CD37 expression by RAMOS and REC1 cells was determined by flow cytometry. Cells were harvested in 2% FCS in phosphate-buffered saline (PBS) and kept on ice prior to use. NNV003 and non-specific human IgG control molecule (Nanogam^®^, Sanquin) were diluted with 2% FCS in PBS to 20 µg/mL and incubated with 2 × 10^5^ cells/mL for 1 h at 4 °C. Bound NNV003 and control antibodies were detected using a phycoerythrin-conjugated goat anti-human IgG secondary antibody (SouthernBiotech; 2040-09) diluted 1:50 with 2% FCS in PBS and analyzed on a BD Accuri C6 flow cytometer (BD Biosciences). Data analysis was performed using FlowJo v10 (Tree Star) and surface receptor expression was expressed as mean fluorescent intensity (MFI).

### Radiolabeling of NNV003 and IgG control for animal studies

NNV003 antibody (IgG_1_, mouse variable regions, κ, and human constant region, κ; Nordic Nanovector) was conjugated to TFP-*N*-sucDf (ABX GmbH) and subsequently radiolabeled with ^89^Zr as described previously^22^. To date, several ^89^Zr-labeled antibodies are produced according to this methodology and were evaluated in animals and patients without any sign of toxicity^16,17,23,24^. In short, NNV003 was incubated with a twofold molar excess of TFP-*N*-sucDf at pH 9.0–9.5. After incubation for 1 h at room temperature (RT), pH was set to 4.0–4.5. Ethylenediaminetetraacetic acid (EDTA; Hospital Pharmacy UMCG) 25 mg/mL was added and incubated for 30 min at 35 °C to transchelate Fe(III) from the TFP-*N*-sucDf hydroxamate groups. NNV003-*N*-sucDf was subsequently purified using a Vivaspin-2 concentrator (Sartorius GmbH), aliquoted and stored at − 80 °C until use. On the day of tracer injection, NNV003-*N*-sucDf was radiolabeled using GMP-grade ^89^Zr oxalate (Perkin Elmer). RCP of [^89^Zr]Zr-*N*-sucDf-NNV003 was determined by trichloroacetic acid precipitation test^17^. Furthermore, NNV003 antibody was conjugated to p-SCN-Bn-DOTA (Macrocyclics) and subsequently radiolabeled using ^177^Lu chloride (Perkin Elmer) as described previously^4,5^.

Non-specific IgG control molecule was conjugated with a 50-fold molar excess of p-SCN-Bn-DTPA (Macrocyclics) as described previously^25^. Radiolabeling of IgG-DTPA was performed using ^111^In chloride (Mallinckrodt) by incubation during 1–2 h in ammonium acetate pH 5.5. Radiochemical purity of [^111^In]In-DTPA-IgG was assessed by instant thin-layer chromatography using 0.1 M citrate buffer pH 6.0 as eluent.

### [^89^Zr]Zr-***N***-sucDf-NNV003 quality control

[^89^Zr]Zr-*N*-sucDf-NNV003 purity and concentration were determined by size-exclusion high-performance liquid chromatography (SE-HPLC). A Waters SE-HPLC system was equipped with a dual-wavelength absorbance detector, in-line radioactivity detector and TSK-Gel SW column G3000SWXL 5 µm, 7.8 mm (Joint Analytical Systems GmbH). PBS (9.0 mM sodium phosphate, 1.3 mM potassium phosphate, 140 mM sodium chloride, pH 7.2; Hospital Pharmacy UMCG) was used as mobile phase at a flow of 0.7 mL/min.

NNV003-*N*-sucDf IRF was determined on human CD37-expressing Burkitt's lymphoma RAMOS cells. Cells were harvested in PBS with 0.5% bovine serum albumin (BSA), diluted to 75 × 10^6^ cells and 0.2 mL added per tube to a total of 5 tubes. CD37-specific binding sites were blocked in 2 tubes by incubation with 20 µg NNV003-*N*-sucDf for 15 min at RT. Subsequently, 8 ng [^89^Zr]Zr-*N*-sucDf-NNV003 (~ 9000 counts per minute) was added to each tube and incubated for 1 h at RT. Tubes were counted in a calibrated well-type gamma counter (LKB instruments), subsequently spun down and washed with 0.5% BSA in PBS for three times, after which tubes were counted again. IRF was expressed as the average percentage of CD37-bound [^89^Zr]Zr-*N*-sucDf-NNV003 as a fraction of the percentage of total activity added in non-blocked tubes corrected for non-specific binding in the blocked tubes. Acceptance criteria were set at ≤ 5% non-specific binding in blocked tubes and NNV003-*N*-sucDf IRF at ≥ 0.8.

### Animal studies

All experiments were performed in accordance with relevant guidelines and regulations. All methods were reported in accordance with recommendations in the ARRIVE guidelines. Animal studies involving [^89^Zr]Zr-*N*-sucDf-NNV003 were approved by the Institutional Animal Care and Use Committee of the University Medical Center Groningen. Male BALB/c OlaHsd-*Foxn1*^*nu*^ mice (Envigo) 8–10 weeks of age were inoculated with 10 × 10^6^ either REC1 or RAMOS cells. Murine NK cells were depleted to enhance tumor take-rate. This was achieved by administering the mice anti-asialo GM1 treatment on 1 day before and 4, 11, 18 and 25 days post tumor inoculation. When tumors measured a volume of at least 200 mm^3^ (~ 14 days post inoculation for REC1 tumors and ~ 19 days for RAMOS tumors), mice received intravenous injections of 10 μg (~ 5 MBq) [^89^Zr]Zr-*N*-sucDf-NNV003 supplemented with either 0, 15 or 90 µg of unlabeled NNV003 and co-injected with an equal total protein dose (~ 1 MBq) of [^111^In]In-DTPA-IgG control (n = 5–6 mice per group). By co-injection of [^89^Zr]Zr-*N*-sucDf-NNV003 and [^111^In]In-DTPA-IgG, tumor uptake and biodistribution results can be compared within the same animal, thereby providing valid results on target-specific uptake. Also, the number of animals required for these studies can be reduced using this strategy. Mice underwent microPET scanning at 1, 3 and 5 days post injection (pi), followed by ex vivo biodistribution.

MicroPET scans were performed using a Focus 220 rodent scanner (CTI Siemens). Scans were reconstructed using a 2-dimensional ordered-subset expectation maximization reconstruction algorithm with Fourier rebinning, 4 iterations, and 16 subsets. Data sets were corrected for decay, random coincidences, scatter, and attenuation. For in vivo quantification, regions of interest were drawn for tumor based upon ex vivo weight, assuming 1 g/cm^3^ tissue density, and heart using AMIDE medical image data examiner software v1.0.4. Tracer uptake was quantified as SUV_mean_ and SUV_max_, calculated from the mean or maximum activity in the region of interest and divided by the injected dose per gram body weight. For ex vivo biodistribution studies, relevant organs were collected, weighed and counted using a calibrated well-type gamma counter. Standards of injected tracer were included to correlate measured counts to the percentage of injected tracer activity. After correction for decay, ex vivo tissue uptake was expressed as the percentage of injected radioactivity dose per gram tissue (%ID/g) and standardized uptake value (SUV) by correcting for injected dose and mouse body weight.

Animal experiments involving [^177^Lu]Lu-DOTA-NNV003 were approved by the Norwegian Animal Research Authority. Biodistribution of [^177^Lu]Lu-DOTA-NNV003 was studied in the RAMOS tumor model. Female Hsd:Athymic Nude-*Foxn1*^*nu*^ mice (Envigo) 7–11 weeks of age were subcutaneously injected with 100 µL RAMOS cell suspension from a donor mouse xenograft to enhance tumor take-rate. Mice received intravenous injections of 4–10 µg (0.5–0.9 MBq) [^177^Lu]Lu-DOTA-NNV003 (IRF 74.4–81.7%), followed by tissue collection and ex vivo biodistribution analysis at 1 h, 6 h, 1 day and 3 days pi (n = 4 mice per group).

### Ex vivo tissue preparation and immunohistochemistry

For ex vivo tissue analysis, formalin-fixed paraffin-embedded (FFPE) tumor tissue blocks were prepared. FFPE blocks were sliced into 4 µm tumor tissue sections, fixated on microscope slides and dried overnight at 60 °C. For CD37 immunohistochemistry, tumor tissue sections were deparaffinized in xylene and rehydrated. Heat-induced antigen retrieval was performed in 10 mM citrate (pH 6.0) for 15 min at 95–100 °C. Endogenous peroxidase was blocked by 10-min incubation with 10% hydrogen peroxide in PBS. Slides were incubated with rabbit anti-human CD37 antibody (Proteintech; 21044-1) or rabbit IgG antibody control (Abcam; ab172730) diluted to 0.8 µg/mL in 1% BSA in PBS for 1 h at RT. Thereafter, slides were incubated with Dako EnVision horseradish peroxidase system (Agilent Technologies) for 30 min at RT, followed by 10-min incubation with diaminobenzidine chromogen. Hematoxylin counterstaining was applied routinely. For histological analysis of tumors, hematoxylin/eosin staining was performed on subsequent tissue sections. Digital scans of slides were acquired by a Hamamatsu NanoZoomer 2.0-HT multi-slide scanner and analyzed with NanoZoomer Digital Pathology viewer software.

### Production and stability testing of clinical grade [^89^Zr]Zr-N-sucDf-NNV003

NNV003 was conjugated to TFP-N-sucDf at a 1:2 molar ratio and subsequently radiolabeled with GMP-grade ^89^Zr. Quality control was performed on both NNV003-*N*-sucDf intermediate product and [^89^Zr]Zr-*N*-sucDf-NNV003 final product. This included analysis on appearance, yield, purity, concentration, pH, radiochemical purity, residual solvents, sterility, endotoxin content and IRF. Analytical procedures were validated to demonstrate suitability for use in quality control testing of NNV003-*N*-sucDf and [^89^Zr]Zr-*N*-sucDf-NNV003. The production processes for NNV003-*N*-sucDf and [^89^Zr]Zr-*N*-sucDf-NNV003 were validated according to GMP guidelines by the production of three consecutive validation batches.

NNV003-*N*-sucDf intermediate product was stored in sterile vials (BioPure) at − 80 °C. Stability of NNV003-*N*-sucDf was analyzed at 0, 1, 3 and 6 months after production. [^89^Zr]Zr-*N*-sucDf-NNV003 final product was stored in sterile vials (BioPure) at 2–8 °C and stability was analyzed at 0 and 96 h. Stability of [^89^Zr]Zr-*N*-sucDf-NNV003 in the syringe at RT was analyzed at 0 and 4 h. Stability tests consisted of quality control according to release specifications.

### Statistical analysis

Data were analyzed for statistical significance in GraphPad Prism v7.0 using the Mann–Whitney U test for non-parametric data followed by Bonferroni post-test correction for comparison of more than two groups. Ex vivo biodistribution of [^89^Zr]Zr-*N*-sucDf-NNV003 and [^177^Lu]Lu-DOTA-NNV003 were compared with Welch’s t-test for unequal variances. Correlation was assessed by Spearman’s rank-order correlation test. In vitro experiments were repeated at least 3 times. *p* values < 0.05 were considered significant.

## Supplementary Information

Supplementary Information.

## Data Availability

Data that support the findings of this study are available from E. G. E. de Vries upon request.

## Abbreviations

^68^Ga: Gallium-68
^111^In: Indium-111
^131^I: Iodine-131
^90^Y: Yttrium-90
^89^Zr: Zirconium-89
BSA: Bovine serum albumin
CI: Confidence interval
CT: Computed tomography
EDTA: Ethylenediaminetetraacetic acid
FCS: Fetal calf serum
FFPE: Formalin-fixed paraffin-embedded
GMP: Good manufacturing practice
HAMAs: Human-anti-mouse antibodies
ID: Injected dose
IRF: Immunoreactive fraction
MFI: Mean fluorescent intensity
PBS: Phosphate buffered saline
PET: Positron emission tomography
pi: Post injection
RCP: Radiochemical purity
RIT: Radioimmunotherapy
RT: Room temperature
SE-HPLC: Size-exclusion high-performance liquid chromatography
SD: Standard deviation
SPECT: Single-photon emission computed tomography
SUV: Standardized uptake value
SUV_mean_: Mean standardized uptake value
SUV_max_: Maximum standardized uptake value
TFP-*N*-sucDf: Tetrafluorphenol-*N*-succinyldesferal
