# Supplementary material for: 89Zr-PET imaging to predict tumor uptake of 177Lu-NNV003 anti-CD37 radioimmunotherapy in mouse models of B cell lymphoma
Source: Sci Rep. 2022 Apr 15;12:6286. doi: 10.1038/s41598-022-10139-6 (PMC9012778; doi:10.1038/s41598-022-10139-6)
Supplement: Supplementary file 1 — Supplementary Information. [file 41598_2022_10139_MOESM1_ESM.docx]

**Supplementary materials**

**
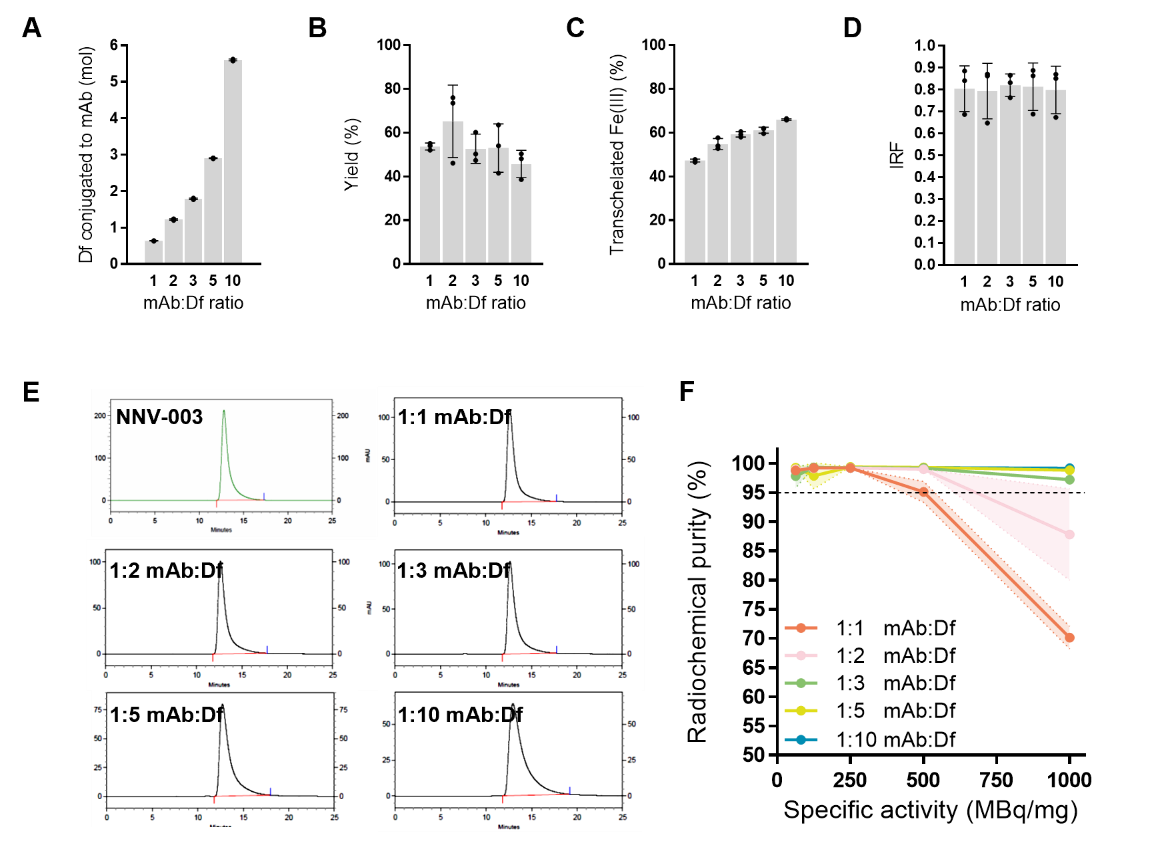
**

**Fig. S1:** Quality control of ^89^Zr[Zr]-N-sucDf-NNV003 production for *in vivo* studies.

**A** Efficiency of NNV003 conjugation to TFP-N-sucDf. The NNV003 to TFP-N-sucDf (mAb:Df) ratio is presented on the x-axis, the effective amount of TFP-N-sucDf chelator conjugated per NNV003 antibody is presented on the y-axis. **B** NNV003 yield after conjugation to increasing molar ratios of TFP-N-sucDf. The mAb:Df ratio is presented on the x-axis, percentage yield after conjugation is presented on the y-axis. **C** Efficiency of Fe(III) transchelation from TFP-N-sucDf hydroxamate groups to EDTA. The mAb:Df ratio is presented on the x-axis, percentage of total Fe(III) transchelated to EDTA is presented on the y-axis. **D** ^89^Zr[Zr]-N-sucDf-NNV003 immunoreactivity to CD37 after conjugation and radiolabeling. The mAb:Df ratio is presented on the x-axis and NNV003-N-sucDf immune reactive fraction (IRF) is expressed on the y-axis. **E** Representative HPLC chromatograms showing purity and aggregates after conjugation of NNV003 to increasing molar excess of TFP-N-sucDf. **F** Radiochemical purity of ^89^Zr[Zr]-N-sucDf-NNV003 at increasing specific activity. Amount of ^89^Zr in MBq added to 1 mg of NNV003 is presented on the x-axis, effective amount of ^89^Zr labeled to NNV003 as percentage of total added radioactivity is presented on the y-axis. Data in A-D and F is shown as mean ± SD.


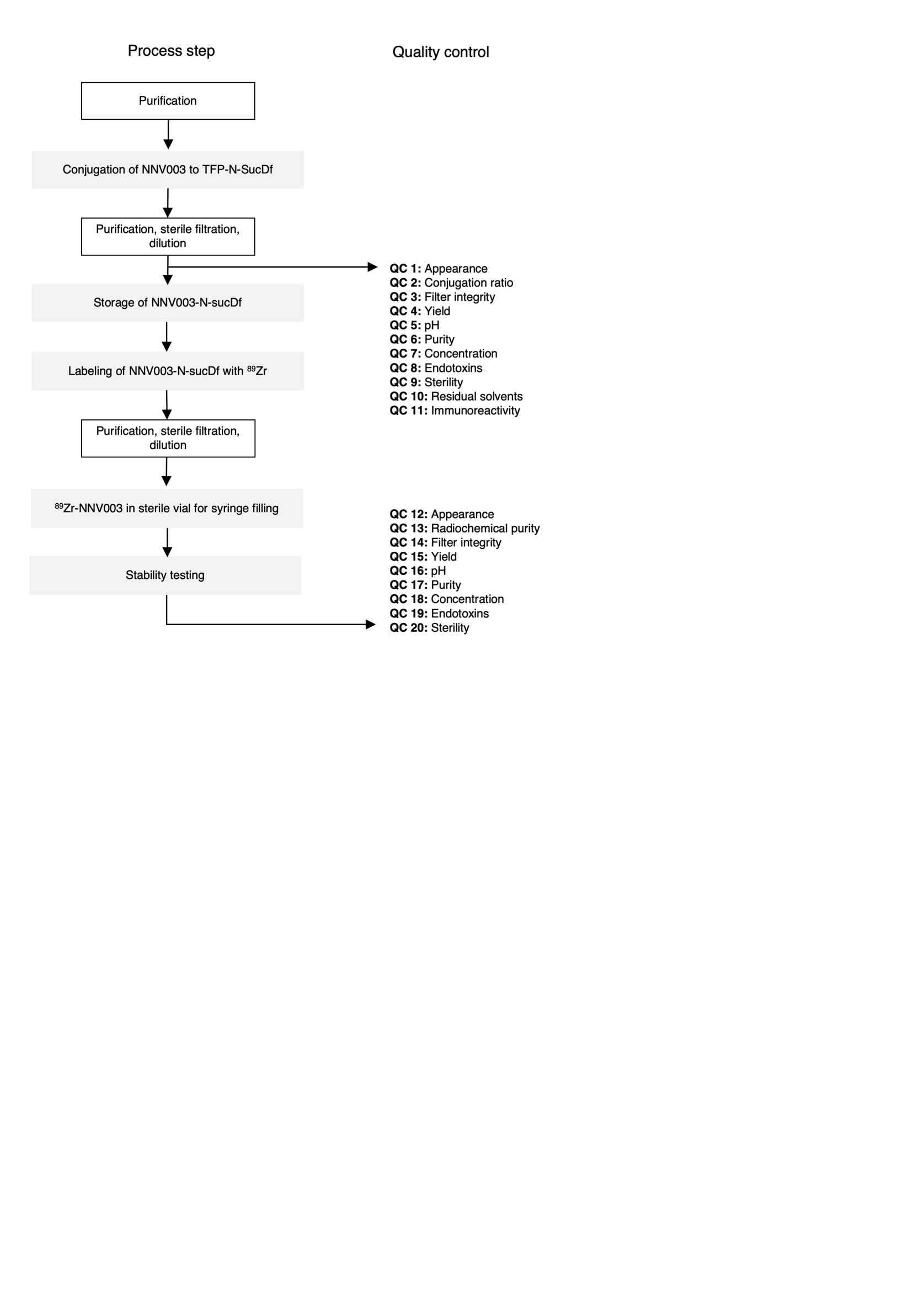


**Fig. S2:** Production process for clinical-grade ^89^Zr[Zr]-N-sucDf-NNV003.

Steps-wise process for the production of good manufacturing practice (GMP)-compliant ^89^Zr[Zr]-N-sucDf-NNV003. Quality control (QC) was performed on NNV003-N-sucDf intermediate product (QC 1-11) and ^89^Zr[Zr]-N-sucDf-NNV003 final product (QC 12-20).

**Table S1:** Quality control of clinical-grade NNV003-N-sucDf and ^89^Zr[Zr]-N-sucDf-NNV003.

| Test | Specification | Batch 1 | Batch 2 | Batch 3 |
| --- | --- | --- | --- | --- |
| *NNV003-N-sucDf intermediate product* | | | | |
| Appearance | Colorless to light yellow | Colorless to light yellow | Colorless to light yellow | Colorless to light yellow |
| Conjugation ratio | 0.5 – 2.0 | 1.47 | 1.41 | 1.39 |
| Filter integrity | < 20% | < 20% | < 20% | < 20% |
| Yield | > 50% | 82.9% | 92.5% | 89.9% |
| pH | 4.0 – 7.0 | 5.34 | 5.23 | 5.30 |
| Purity | ≤ 3% aggregates (280 nm) | < 3% | < 3% | < 3 % |
| Concentration | 9.0 – 11.0 mg/mL | 9.7 mg/mL | 9.6 mg/mL | 9.8 mg/mL |
| Endotoxins | < 2.5 EU/ml | < 2.5 EU/mL | < 2.5 EU/mL | < 2.5 EU/mL |
| Sterility | Sterile | Sterile | Sterile | Sterile |
| Residual solvents  (acetonitrile) | < 410 ppm | < 410 ppm | < 410 ppm | < 410 ppm |
| IRF | 50 – 100% | 86.8% | 67.8% | 64.6% |
| *^89^Zr[Zr]-N-sucDf-NNV003 final product* | | | | |
| Appearance | Colorless to light yellow | Colorless to light yellow | Colorless to light yellow | Colorless to light yellow |
| RCP | ≥ 95% | 99.1% | 99.3% | 99.4% |
| Filter integrity | < 20% | < 20% | < 20% | < 20% |
| Yield | For 1 patient  > 40.7 MBq  For 2 patients  > 77.7 MBq | 49.77 MBq | 64.03 MBq | 80.28 MBq |
| pH | 5.0 – 8.0 | 5.07 | 5.75 | 5.76 |
| Purity | ≤ 3% aggregates (280 nm) | < 3% | < 3% | < 3 % |
| Concentration | For information only | 0.15 mg/mL | 0.10 mg/mL | 0.07 mg/mL |
| Endotoxins | < 2.5 EU/ml | < 2.5 EU/mL | < 2.5 EU/mL | < 2.5 EU/mL |
| Sterility | Sterile | Sterile | Sterile | Sterile |

Quality control results for three individual batches of NNV003-N-sucDf intermediate product and ^89^Zr[Zr]-N-sucDf-NNV003 final product (37 MBq to ~1 mg). IRF was determined after labeling with ^89^Zr. EU: endotoxin units, IRF: immune reactive fraction, RCP: radiochemical purity, ppm: parts per million.

**Table S2:** Stability of clinical-grade NNV003-N-sucDf.

| Test | Specification | t = 0 | 1 month | 3 months | 6 months |
| --- | --- | --- | --- | --- | --- |
| Appearance | Colorless to light yellow | Colorless to light yellow | Colorless to light yellow | Colorless to light yellow | Colorless to light yellow |
| pH | 4.0 – 7.0 | 5.34 | 5.36 | 5.33 | 5.31 |
| Purity | ≤ 3% aggregates (280 nm) | ≤ 3% | ≤ 3% | ≤ 3% | ≤ 3% |
| Concentration | 9.0 – 11.0 mg/mL | 9.7 mg/mL | 9.4 mg/mL | 9.5 mg/mL | 9.3 mg/mL |
| Endotoxins | < 2.5 EU/ml | < 2.5 EU/mL | nd | nd | nd |
| Sterility | Sterile | Sterile | nd | nd | nd |
| Residual solvents  (acetonitrile) | < 410 ppm | < 410 ppm | nd | nd | nd |
| RCP | ≥ 95% | 99.1% | 99.2% | 99.3% | 99.6% |
| Immunoreactivity | 50 – 100% | 86.8% | 69.3% | 79.4% | 82.2% |

Quality control results for stability of NNV003-N-sucDf intermediate product (batch 1) at 1, 3 and 6 months. EU: endotoxin units, IRF: immune reactive fraction, nd: not determined, RCP: radiochemical purity, ppm: parts per million.

**Table S3:** Stability of clinical-grade ^89^Zr[Zr]-N-sucDf-NNV003.

| Test | Specification | t = 0 | 96 h (2 – 8 °C) | 4 h syringe (RT) |
| --- | --- | --- | --- | --- |
| Appearance | Colorless to light yellow | Colorless to light yellow | Colorless to light yellow | Colorless to light yellow |
| pH | 5.0 – 8.0 | 5.76 | 5.89 | 5.83 |
| Purity | ≤ 3% aggregates (280 nm) | ≤ 3% | ≤ 3% | ≤ 3% |
| Concentration | For information only | 0.07 mg/mL | 0.07 mg/mL | 0.06 mg/mL |
| Endotoxins | < 2.5 EU/ml | < 2.5 EU/mL | nd | nd |
| Sterility | Sterile | Sterile | Sterile | Sterile |
| RCP | ≥ 95% | 99.4% | 97.9% | 97.8% |

Quality control results for stability of ^89^Zr[Zr]-N-sucDf-NNV003 final product (produced from batch 3 of NNV003-N-sucDf intermediate product) for 96 h at 2-8 °C and for 4 h in the syringe at RT. EU: endotoxin units, nd: not determined, RCP: radiochemical purity, RT: room temperature, ppm: parts per million.
